# Predicting the environmental suitability for *Anopheles stephensi* under the current conditions in Ghana

**DOI:** 10.1038/s41598-024-51780-7

**Published:** 2024-01-11

**Authors:** Rahmat Bint Yusif Ismail, Faramarz Bozorg-Omid, Joseph Harold Nyarko Osei, Sellase Pi-Bansa, Kwadwo Kyeremeh Frempong, Mavis Koryo Ofei, Helena Anokyewaa Boakye, Jane Ansah-Owusu, Sandra-Candys Adwirba Akorful, Christopher Nii Laryea Tawiah-Mensah, Mufeez Abudu, Andy Asafu-Adjaye, Maxwell Alexander Appawu, Daniel Adjei Boakye, Hassan Vatandoost, Mohammad Mehdi Sedaghat, Fahimeh Youssefi, Ahmad Ali Hanafi-Bojd, Samuel Kweku Dadzie

**Affiliations:** 1 Department of Vector Biology and Control of Diseases, School of Public Health, Tehran University of Medical Sciences, Tehran, Iran; 2 Department of Parasitology, Noguchi Memorial Institute for Medical Research, College of Health Sciences, University of Ghana, Legon, Ghana; 3 Department of Photogrammetry and Remote Sensing, K. N. Toosi University of Technology, Tehran, Iran; 4 Zoonoses Research Center, Tehran University of Medical Sciences, Tehran, Iran

**Keywords:** Ecological modelling, Invasive species

## Abstract

Vector-borne diseases emergence, particularly malaria, present a significant public health challenge worldwide. Anophelines are predominant malaria vectors, with varied distribution, and influenced by environment and climate. This study, in Ghana, modelled environmental suitability for *Anopheles stephensi*, a potential vector that may threaten advances in malaria and vector control. Understanding this vector’s distribution and dynamics ensures effective malaria and vector control programmes implementation. We explored the MaxEnt ecological modelling method to forecast *An. stephensi*’s potential hotspots and niches. We analysed environmental and climatic variables to predict spatial distribution and ecological niches of *An. stephensi* with a spatial resolution of approximately 5 km^2^. Analysing geospatial and species occurrence data, we identified optimal environmental conditions and important factors for its presence. The model’s most important variables guided hotspot prediction across several ecological zones aside from urban and peri-urban regions. Considering the vector’s complex bionomics, these areas provide varying and adaptable conditions for the vector to colonise and establish. This is shown by the AUC = 0.943 prediction accuracy of the model, which is considered excellent. Based on our predictions, this vector species would thrive in the Greater Accra, Ashanti Central, Upper East, Northern, and North East regions. Forecasting its environmental suitability by ecological niche modelling supports proactive surveillance and focused malaria management strategies. Public health officials can act to reduce the risk of malaria transmission by identifying areas where mosquitoes may breed, which will ultimately improve health outcomes and disease control.

## Introduction

Malaria is a potentially fatal infection that is transmitted to people through the bites of infected female *Anopheles* mosquitoes. Nearly half of the world's population was at risk of malaria in 2021^1^. COVID-related interruptions increased malaria incidence and fatalities at the pandemic's peak (2020–2021)^2^. Sub-Saharan Africa has the highest global malaria mortality and morbidity rates. As of 2021, the WHO African Region recorded approximately 95%, of all reported malaria cases and 96% of deaths^1–3^. Despite significant efforts to scale up vector control strategies, including the use of Long-Lasting Insecticidal Nets (LLINs) and Indoor Residual Spraying (IRS), this high rate of malaria persists. This is a direct effect of the continent's highly efficient *Anopheles* vector species, *Anopheles gambiae* complex^4^.

*Anopheles stephensi* species, an Asian malaria vector, has become an invasive species in the Horn of Africa (HOA) in recent years^5,6^. It was initially discovered on the African continent in 2012 in a Djibouti seaport, then in neighbouring Ethiopia in 2018, and 2019 near Sudan seaports, and Somalia in 2019^6,7^. Given the species' distinctive biological traits and identification in seaports, it has been predicted that their emergence was most likely helped by maritime commerce^6^. This invasive *An. stephensi* population, which has successfully established itself in several African countries, represents a new challenge to malaria control and elimination on the African continent^8,9^. The ability of this vector to adapt and thrive in urban surroundings could undermine malaria control and elimination efforts. In contrast to indigenous African mosquitoes, this Asian malaria vector is one of the unique *Anopheline* species present in major metropolitan areas^9,10^.

The World Health Organization (WHO) issued a vector alert in 2019 encouraging nations in Africa to act promptly to improve vector surveillance to monitor and stop the spread of the *An. stephensi* vector^1,7^. Ghana is one of the African countries where malaria is endemic and perennial in all parts of that with seasonal variations more pronounced in the north^11^. There is the threat of establishing invasive *An. stephensi* vector in Ghana that was recently discovered in some areas of urban (Greater Accra) in Ghana^1^. It is possible that *An. stephensi* has been inadvertently introduced into Ghana multiple times, but more research is needed to verify this. In areas where invasive species are expanding their ranges to new areas, there is a need to quickly identify these areas to slow down or eliminate this invasion^12^.

Species distribution models (SDMs), also known as Ecological Niche Models (ENMs), are very useful tools for identifying distributions and environmental suitability of invasive species^13^. These models can be used to understand the responses of invasive species to climatic variables. In other words, these models use occurrence points of species and environmental data to predict their habitats with a high probability of the presence^14^. Some approaches have been developed over the last decade in a review study, it was found that more than 35 modelling approaches have been developed for generating SDMs. The most common models in the world are Maximum Entropy (MaxEnt), Generalized Linear Model (GLM), Random Forest (RF), and Generalized Boosting Model (GBM)^15^. Recently, studies comparing several of these approaches indicated that the MaxEnt model performed as well or better than the other approaches. As such, a developed MaxEnt model is a great potential tool for determining suitability environmental given its reliance on only presence locations^16–18^.

The purpose of this study was to model the current environmental suitability for *An. stephensi* and predict hotspot areas in Ghana to implement surveillance strategies and create effective management and surveillance strategies for this vector. This is crucial for Ghana's vector and malaria control programs to be effective and successful.

## Results

### Environmental variables selected

**Figure 1:**
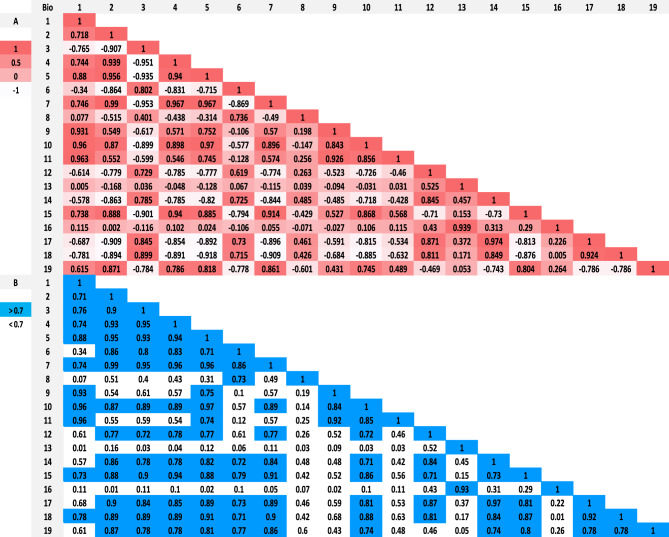
(**A**) Absolute value of (r) and (**B**) Pearson correlation coefficient (r) for climatic variables.

The bioclimatic variables had high correlations, based on Pearson's correlation Coefficient (PCC) values (Fig. 1). Any two variables that had a correlation coefficient of |r|≥ 0.7 were deemed to be highly associated. Following the PCC statistical analysis of the outcomes, a subset of eight variables consisting of four environmental variables and four climatic layers: elevation, slope, Normalized Difference Vegetation Index (NDVI), population, Annual mean temperature (°C) (Bio1), Minimum temperature of the coldest month (°C) (Bio6), Annual precipitation (mm) (Bio12), and Precipitation of wettest month (mm) (Bio13) were used for the species distribution prediction modelling of the target species (*An. stephensi*) (Table 1).

**Table 1:** Bioclimatic and environmental variables were used in the model.

Variables	Description	Percent contribution	Permutation importance
Bio 1	Annual mean temperature (°C)	21.3	55.5
Bio 6	Minimum temperature of coldest month (°C)	2.3	8.5
Bio 12	Annual precipitation (mm)	5.8	1.4
Bio 13	Precipitation of wettest month (mm)	0.9	3.4
Elevation	Altitude (m)	0.9	1.6
Slope	Gradient or rate of maximum change in Z-value	2.5	1.3
NDVI	Normalized Difference Vegetation Index	1.1	0.4
Population	Population density	65.2	28

Source: Altitude and bioclimatic data were downloaded from the WorldClim (v2.1) database, www.worldclim.org; slope and NDVI layers were obtained from Google Earth Engine (Modis satellite images); and population density grid downloaded from the website of socioeconomic data and application center.

### Model performance

**Figure 2:**
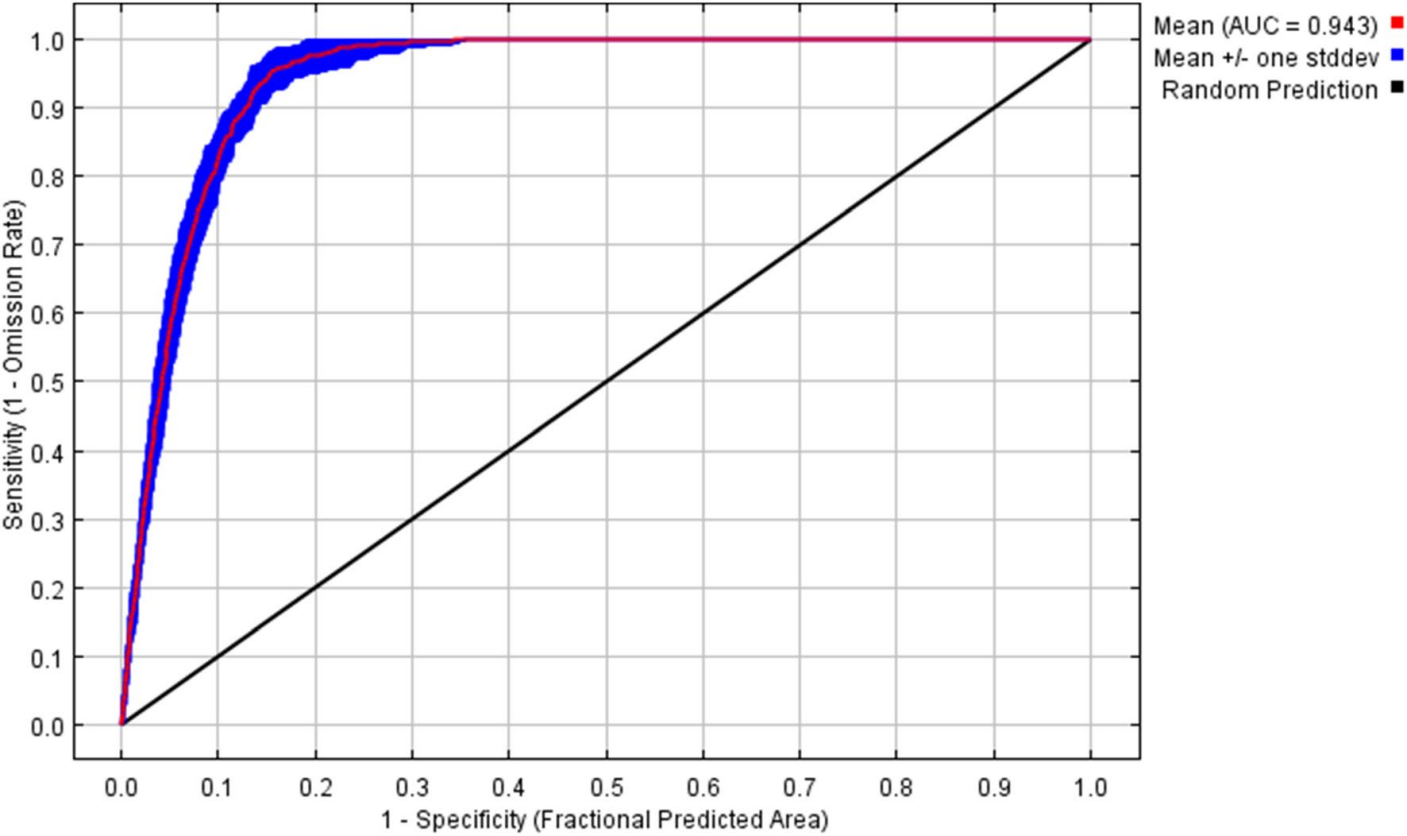
Receiver operating characteristic curve.

The Receiver Operating Characteristic curve (ROC) determines the AUC's ability to evaluate the predictive ability of the model. The final model's AUC mean over ten iterations was 0.943, and the standard deviation was 0.008 (Fig. 2). This demonstrates the model's strong functionality and great prediction accuracy. *An. stephensi*’s prospective global distribution areas can be accurately predicted using the species' distribution and environmental factors data used in this modelling.

### Current distribution of suitable habitat

**Figure 3:**
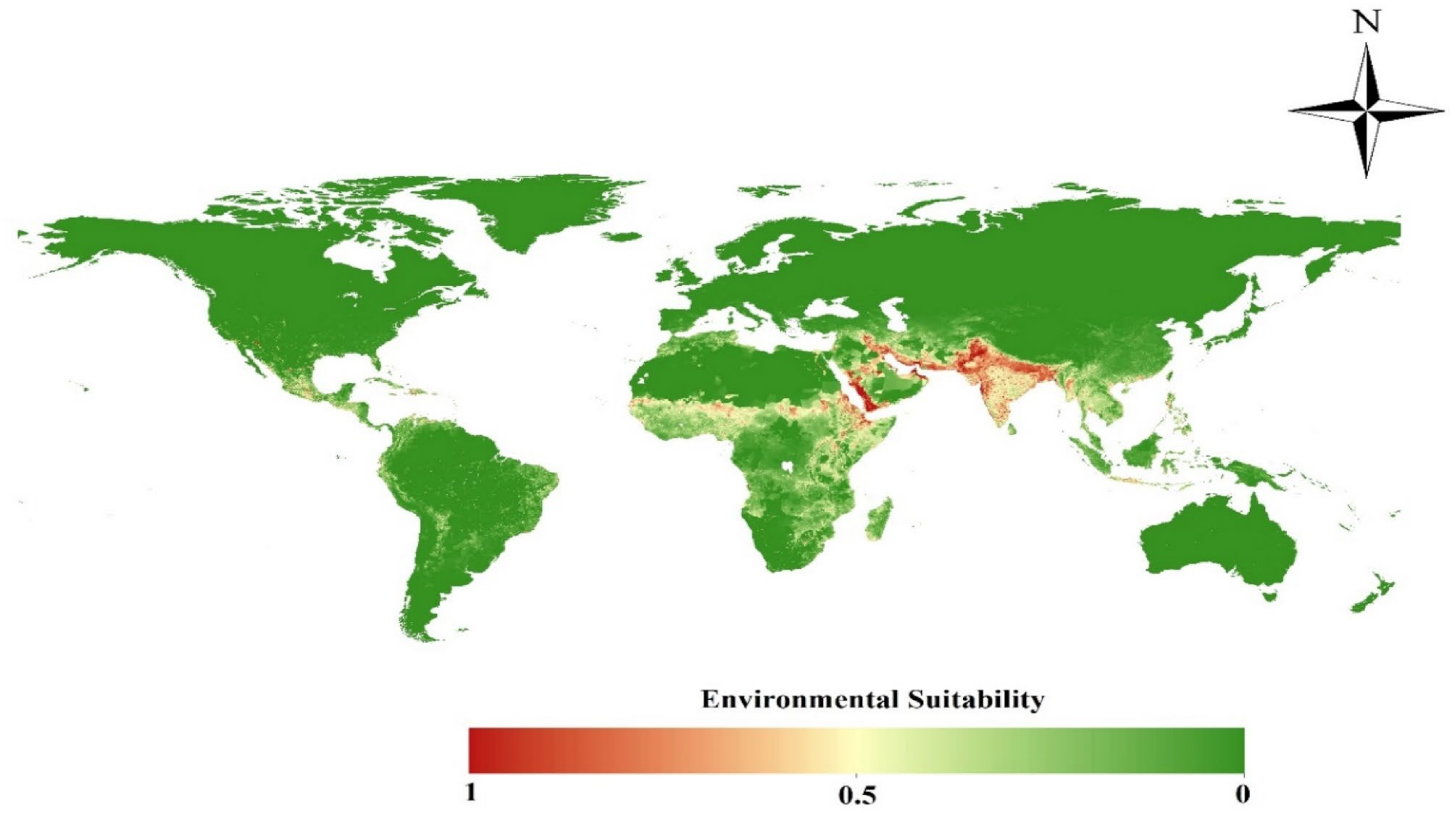
Environmental suitability of the *Anopheles stephensi* species under current climatic conditions in the world. The Map was generated using ArcGIS v10.5 (www.esri.com).

**Figure 4:**
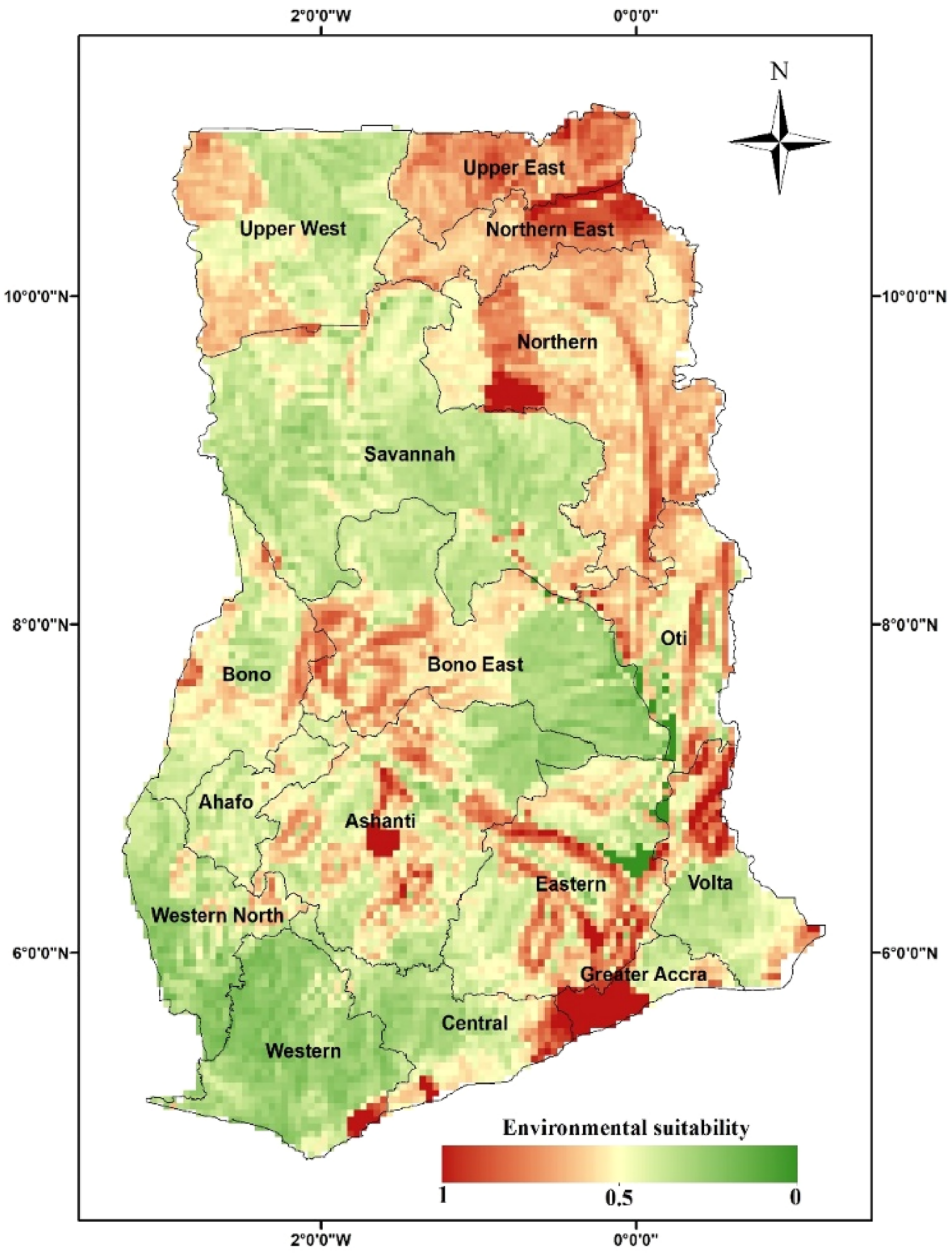
Environmental suitability of the *Anopheles stephensi* species under current climatic conditions in Ghana. The Map was generated using ArcGIS v10.5 (www.esri.com).

The MaxEnt model's output of *An. stephensi*-friendly environments around the globe are shown with a spatial resolution of approximately 5 km^2^ in Fig. 3. The Arabian Peninsula and South-East Asia, where the *An. stephensi* species is native and is projected to have the best circumstances for its dispersion. However, the most favourable conditions can be found in the areas where it has invaded, which stretch from the southernmost point of the European continent to sections of the Middle East, the Horn of Africa, Eastern and Central Africa, and West Africa. In several areas of Central and South America, suitable conditions for the species' dispersion are also predicted (Fig. 3). The ecological niches for *An. stephensi* in Ghana has expanded from the north-eastern (Upper East) to the southern (Greater Accra) regions of the country under the current conditions. Furthermore, the Ashanti and Bono East regions in the middle belt are projected to have a significant possibility of having this vector present (Fig. 4).

### Species response and potential habitat suitability distribution

**Figure 5:**
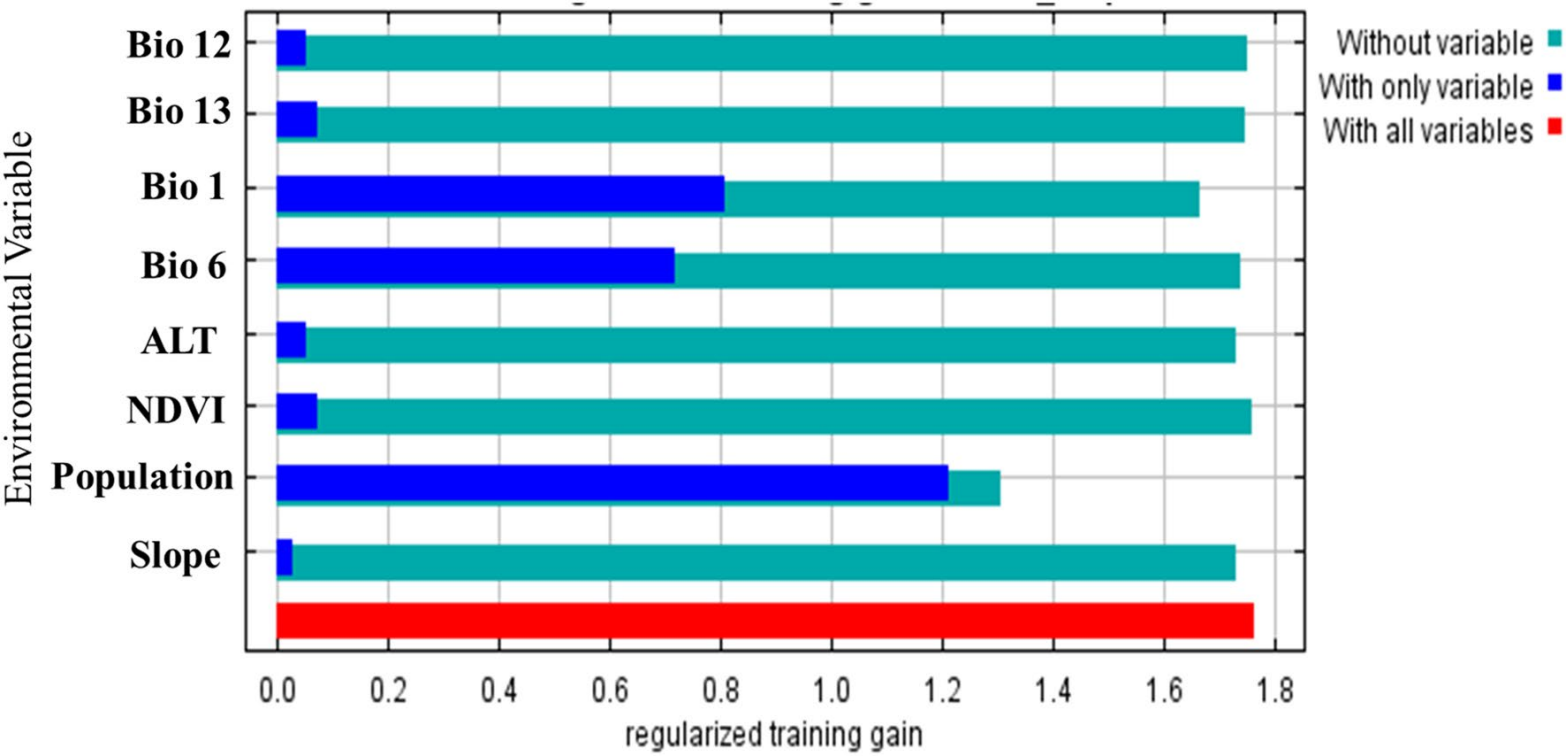
Jackknife of Regularized Training Gain for *Anopheles stephensi*.

To get estimates of which variables are most important in the model, we used the jackknife analysis in the MaxEnt model (Fig. 5). The population appeared to have the most useful information by itself because it was the environmental variable that gained the most when used alone. In other words, the population variable allows a reasonably good fit to the training data. It also appears to provide the most information that is not contained in the other variables because it was the variable that reduced the gain the greatest when it was omitted (Fig. 5). These findings demonstrate that population and yearly mean temperature (Bio1) contributions were 65.2% and 21.3%, respectively, and that regularized training gain values were both more than 0.75, which could be more beneficial for the prediction model (Fig. 5). Population and Bio1, thus offered more insightful data than the other environmental and climatic factors and had a greater impact on the global distribution of the *An. stephensi* species. In other words, the evaluation of the variables' contributions reveals that population density was mostly used in the modelling, whereas Bio1 and population variable earned the greatest permutation importance (55.5 and 28%, respectively), showing its ability to predict the outcome of the model when used alone (Table 1).

**Figure 6:**
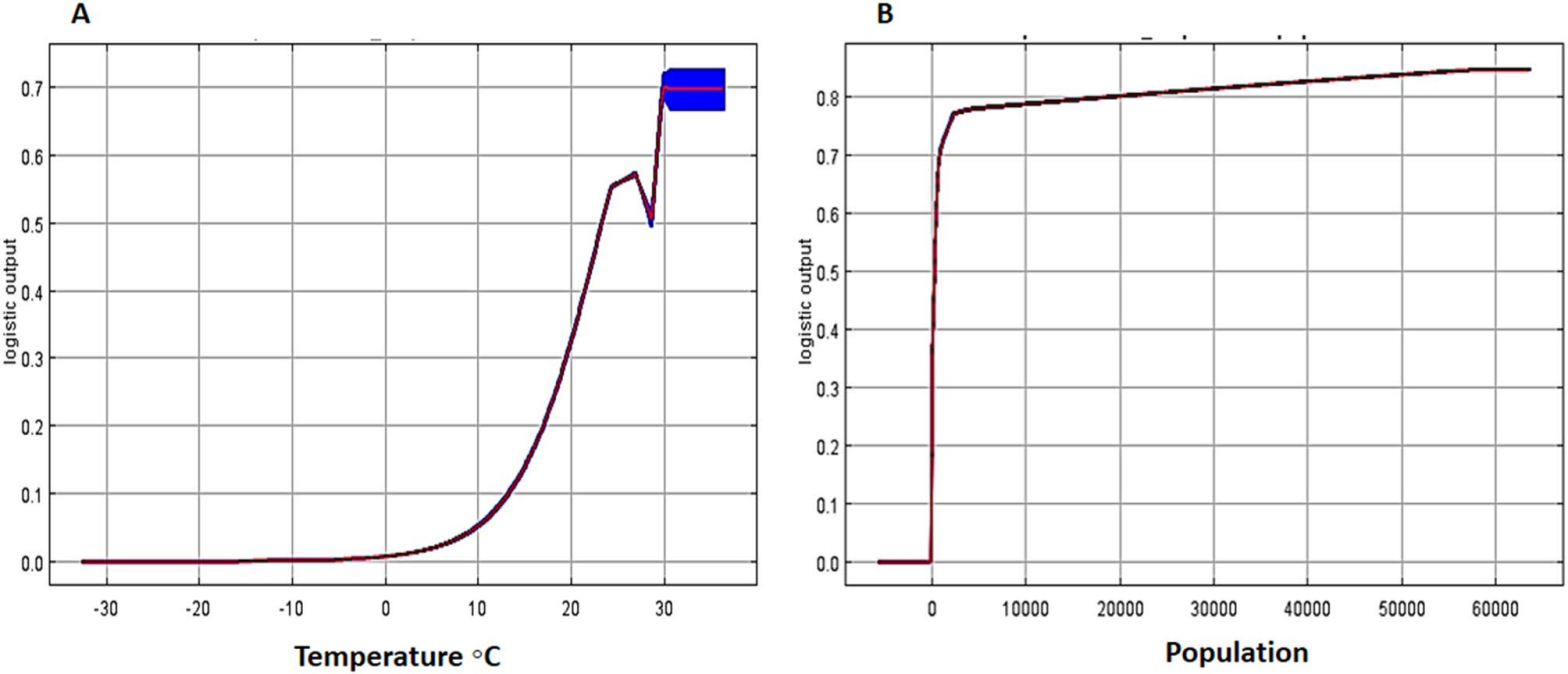
Response curves of the (**A**) annual mean temperature (Bio 1) (**B**) population variables that contributed the most the MaxEnt models. The curves show the mean response of the 10 replicate MaxEnt runs (red) and the mean + /− one standard deviation.

The program produced a second set of response curves, in which each curve is made by generating a model using only the corresponding variable, disregarding all other variables (Fig. 6). In other words, Fig. 6 describes the response curve for the primary environmental factor influencing the spread of *An. stephensi*. The value shown on the y-axis is predicted probability of suitable conditions, as given by the logistic output format. The curve demonstrates that only the relevant variable (Bio1) was used to develop the MaxEnt model. The air temperature response curve's annual mean temperature change exhibits a general upward pattern between 5 and 30 °C. The presence probability will be established when the yearly mean air temperature change exceeds 30 °C, where the maximum probability of presence is 70%. Inferring that the species is more sensitive to variations in annual mean temperature is possible (Fig. 6A). Furthermore, the curves show how the predicted probability of presence changes with population variable is varied. As the population increases, the probability of the presence of the *An. stephensi* is increasing (Fig. 6B).

### Entomological study

Morphological identification and pictures were conducted using the Motic Plus microscope series. A total of 905 mosquito specimens from the collected larvae emerged and were morphologically identified. Out of this number, 720 were identified as *An. gambiae s.l.*, 146 identified as *Culex* spp., (mostly *Cx. quinquefasciatus*) 4 identified as *Aedes* spp. (*Ae. aegypti*) and 35 were unidentified. Because the diagnostic characteristics for identification of some samples were damaged, we listed them as “unidentified” samples. No specimen was morphologically identified as *An. stephensi.* (Table 2).

**Table 2:** Collection sites visited for *Anopheles stephensi* and the various mosquito species collected, Ghana, April–May, 2023.

Study site (major towns)	Location	GPS coordinates		Mosquito species caught				
		Longitude	Latitude	*Anopheles gambiae*	*Anopheles stephensi*	*Aedes* spp.	*Culex* spp.	Unidentified
Adenta East	Gogo Street	−0.14840	5.71520	5	0	0	3	0
	Housing Down	−0.14565	5.69842	15	0	0	1	0
	Commando Antoine street	−0.14526	5.72211	2	0	0	3	0
	Lakeside Estate	−0.11653	5.71284	10	0	0	0	1
	New Legon	−0.13206	5.71326	1	0	0	0	0
Sowutoum	Kwashieman Ofankor Road Sp5	−0.27829	5.61648	24	0	0	1	0
	Kolyma River Street Sp1	−0.27165	5.62289	36	0	0	8	1
	Kolyma River Street Sp2	−0.27165	5.62297	20	0	0	0	0
	Tabora junction Sp4	−0.26949	5.62432	19	0	0	3	0
	Tabora junction Sp3	−0.26950	5.62416	3	0	0	0	0
Pokuase	Pokuase fetus	−0.288281	5.695209	24	0	0	0	0
	Spot 1	−0.288939	5.694899	4	0	0	0	1
	Spot 5	−0.261404	5.683593	1	0	0	14	0
	Spot 6	−0.261408	5.682907	13	0	0	4	1
	Spot 3	−0.287654	5.697232	0	0	0	0	0
Gbawe	CP	−0.299925	5.582797	5	0	0	3	0
	Buleme	−0.323757	5.578567	2	0	3	0	0
	Gyama	−0.316834	5.574750	10	0	0	4	0
	Topbase	−0.308462	5.571208	12	0	0	0	0
	Gravel junction	−0.303900	5.578505	5	0	0	0	0
Mandela	Spot 1	−0.332706	5.556941	3	0	0	1	0
	Little falls Sp2	−0.333446	5.560278	18	0	0	56	0
	Black St	−0.340697	5.559738	1	0	0	4	0
	Bing Cherry Sp4	−0.343023	5.558981	0	0	0	1	0
	Spot 5	−0.344594	5.556350	19	0	0	0	1
Kakasunanka	Gbetsile Point 1	−0.01402	5.74280	14	0	0	4	3
	Gbetsile Point 2	−0.02069	5.74423	27	0	0	1	2
	Gbetsile Point 3	−0.02421	5.74272	8	0	0	1	1
	Gbetsile Point 4	−0.02886	5.74388	21	0	0	1	5
	Gbetsile Point 5	−0.03088	5.74398	0	0	0	0	0
Tema New Town	Pentecost Road Sp3	−0.01953	5.65929	64	0	1	2	15
	Old Town Sp2	−0.01980	5.64710	5	0	0	0	1
	Old Town	−0.01986	5.64626	44	0	0	3	3
	Old town Sp1b	−0.02064	5.64546	20	0	0	0	0
	Pentecost Road Sp4	−0.02966	5.66359	27	0	0	0	0
Lashibi	Spot 1	−0.064023	5.645560	8	0	0	8	0
	Tema Village Rd Spot 4	−0.052373	5.619854	31	0	0	3	0
	Spot 3	−0.065718	5.623489	10	0	0	0	0
	Community 16	−0.071052	5.626916	51	0	0	6	0
	Spot 5	−0.058131	5.640715	11	0	0	0	0
Gomoa Fetteh	White Sand	−0.469481	5.429017	1	0	0	0	0
	Sunset Resort	−0.471357	5.414483	0	0	0	2	0
	Till Resort	−0.468553	5.415849	3	0	0	0	0
	Goil Station Sp1	−0.472087	5.432942	0	0	0	0	0
	Hope College	−0.472087	5.432942	0	0	0	0	0
Kasoa	Winneba Road Site 1	−0.41534	5.53772	27	0	0	2	0
	Cowfort Mensah Rd Site 2	−0.43173	5.53630	0	0	0	0	0
	Zain St Site 3	−0.44741	5.53430	7	0	0	6	0
	Accra Rd Site 4	−0.44457	5.52330	43	0	0	0	0
	Kasoa Roundabout Site 5	−0.42485	5.51746	46	0	0	1	0

## Discussion

It seems like *An. stephensi* is spreading to new areas and contributing to outbreaks of urban malaria^9^. Therefore, according to the recently report of WHO, the discovery of the *An. stephensi* vector in Ghana (Greater Accra) is concerning as it is an invasive species that can transmits malaria in Ghana^1^. It's important to monitor and control the spread of this species to prevent further outbreaks of malaria and protect public health^1,7^. Preliminary measures in order to avoid that threat is to predict the possible areas of presence of species^12^. The current suitability environmental of *An. stephensi* was modeled by the MaxEnt model in Ghana in this work. Considering our results, the suitable niches for *An. stephensi* in Ghana have expanded from the north-eastern (Upper East) to the southern (Greater Accra) regions of the country. Furthermore, the Ashanti and Bono East regions in the middle belt are predicted to have favorable areas for the survival of this vector. The mentioned areas are among the urban and densely populated areas of Ghana. On the other hand, Ghana is urbanizing rapidly; more than half of the population now lives in urban areas^19^. Studies have shown that, in contrast to the endemic African mosquitoes, *An. stephensi* is one of the few anopheline species found in central urban locations. This vector is able to thrive in close connotation with people, and thus theoretically able to establish itself everywhere that temperature is not limiting^20^. Our maps and findings in Africa point to a significant future threat to urban African populations. According to a modelling study, if *An. stephensi* were to spread unchecked, 126 million more people in Africa would be in danger of suffering malaria^9^.

After the establishment of this vector and the exposure of the population of Ghana in the future, this country probably will face new challenges. The species is now resistant to all major groups of insecticides as well as developing a variety of resistance mechanisms^21^. Therefore, as its insecticide resistance is widespread, development on new formulations and molecules will be crucial to keep fighting malaria in Ghana. This challenge raises the economic costs of dealing with malaria. It is predicted that if this country does not have the challenges related to malaria control, through malaria elimination, can expect to see a 32-fold return on their investment^11^. The physical changes in *An. stephensi*, specifically its ability to tolerate different temperatures better than *An. gambiae*^22^, are causing concern about its ability to thrive as an invasive species and potentially disrupt efforts to control malaria in Ghana. Another challenge that can be mentioned, the increase in temperature caused by future climate changes in the surrounding environment reduces the time required for the larval stage to mature. As adults, mosquitoes will then digest blood meals at a faster rate. This results in mosquitoes biting humans more frequently and parasites developing more quickly, ultimately leading to an increase in reproduction, efficiency in transmitting malaria, and overall fitness^23,24^.

We also modeled environmental suitability for *An. stephensi* globally. Due to the fact that the modeling was done globally, we had to use lower-resolution data (5 km^2^). Although the resolution of environmental and climatic variables can affect the accuracy of the model output, this effect does not seem to be enough to distort the overall result of the work. Several other studies conducted globally have also used medium-resolution data^25,26^. Our modeling showed that a wide range from Iraq to West China is suitable for its survival. A study recently provided a snapshot of environmental suitability for *An. stephensi* and it demonstrated that India, Pakistan, south of Iran, west of Yemen, Southwest Saudi Arabia, East of Iraq and several countries from the African continent, including Djibouti, Ethiopia are suitable in terms of habitat^9^. In the world scale and as a snapshot the similarity of the modeling results conducted by *Sinka *et al*.* with the outputs of our model shows the consistency of the results. In detail, the whole South of India is overall very suitable for *An. stephensi* (> 0.5), while in our map it is ~ 0.5. West-Africa is marked overall as not suitable (except in the cities), while in our predictions, West-Africa (including Ghana) has a lot of more or less suitable regions (~ 0.5). This differences may be due to differences in the model used^44^. So that, *Sinka *et al*.* ran their models using the biomed2 platform in R studio using R. Moreover, the reference study provided a final set of just seven relevant environmental covariates, refined from an initial set of 19 in their study. Apart from climate, other factors such as topography, and vegetation conditions can affect the distribution of *An. stephensi*. Furthermore, this could be the result of different global climate models (GCMs) that create specific differences in regional climate change prediction. Therefore, we used SSP-MIROC6 in our study. Predictive performance can be impacted by a wide range of variables in addition to the model type, including sample size, spatial scale, environmental variable selection, and the method used to choose pseudo- or absence data^27–29^. The AUC has been widely applied in SDMs and is regarded as the best measurement of predictive power^30,31^. In our analysis, the model was able to predict the distribution of the vector with an excellent level of accuracy (AUC = 0.943). Our findings are in line with those of other studies and support the MaxEnt model's excellent performance^24,32^.

Climate-related factors have an impact on this vector species' distribution, which could influence the incidence of malaria and create outbreaks^33^. Regions with warm temperatures and humid conditions are preferred habitats for *An. stephensi*. The main environmental variable among the eight that affected the suitability of the habitat, and the likelihood of the species distribution was the annual mean temperature^36^. Changes in temperature have a direct impact on the populations of *An. stephensi* and is a critical factor in its habitat and reproduction^37^. This is consistent with research carried out across the globe^38^. When the amount of yearly temperature variation was between 5 and 30 °C, it could generate an effective accumulation temperature for *An. stephensi* when combined with the response curves of environmental factors output from the prediction model. The plot reflect the dependence of the prediction suitability on the selected variable and the dependence caused by the correlation between the selected variable and other variables^39^. According to the response curve, when the annual mean temperature (Bio 1) is between ≈24.0 and 30.0 °C, this is most suitable for the survival of *An. stephensi.* According to the plot, the temperature of ≈26.0 °C and 30.0 °C indicates two suitable peaks in terms of average annual temperature for the survival of *An. stephensi*, and remains stable after 30.0 °C. Studies have reported that this species has two peaks of activity in field conditions, which appear in different months depending on the regions of its presence^20,36^. Artificial habitats produced by human activities inside or on the edge of residential areas are considered to be the most suitable places for spawning and hatching of *An. stephensi*^40–42^. In recent research, based on the study that was conducted, there were many suitable habitats in residential areas^9,43^. These areas are potentially prone to the spawning of *An. stephensi* and action should be taken to improve the environment and reduce suitable areas for the growth and development of this dangerous vector.

Our results added more detail about the presence/absence of *An. stephensi* in Ghana. Although the results from our entomological study indicated no specimens were identified as *An. stephensi*, in a recent report published by the WHO on *An. stephensi,* in Africa, this species was recently reported from two locations in Greater Accra, Ghana^1^. It is interesting to note that based on the output of the model in our study, these two points have been identified as prone to the presence of *An. stephensi* in terms of environmental conditions. The absence of this species in predicted areas can have various reasons. The main factor is that we only collected samples once in this study. As another reason, it’s essential to note that modelling studies aim just to predict a species' habitat suitability. In other words, predicting the areas with the probability of the presence of a certain species does not mean the definite presence of that species^44^. Generally speaking, the report of specimens from Ghana raises some interesting questions. Is *An. stephensi* a recent introduction to the area, or has it been present but simply went undetected due to its morphological similarity to *An. arabiensis*. Further research and investigation will be necessary to shed more light on this issue. Therefore, the probability of the presence of this species in the areas predicted by this model should be taken seriously studies and regular and periodic monitoring in hotspot areas on the *Anopheles* fauna should carried out using the new morphological identification key of African *Anopheles*^45^ as well as molecular methods.

Like any modeling tool, MaxEnt can have limitations that need to be addressed to obtain better results. For example, MaxEnt is influenced by heavily biased sampling distributions, although this bias can be reduced by targeting background locations from sampled areas^46^. There are also a few limitations in this study. First and foremost, MaxEnt is limited to the analysis of abiotic factors, such as temperature and the results output by the model do not consider the influences of biological factors on species distribution^47^. In other words, the predicted output does not always take biological and physical barriers to species movement into consideration. It is vital that future studies take a larger variety of potential variables into account. It would be great to consider the combination of abiotic and biotic factors in future prediction work, and this is an area that deserves attention and reflection in future forecasting efforts. One of the limitations of the study is related to historical climate data. These data have been produced from 1970 to 2000, and due to recent climate changes, the climatic conditions have changed since 2000. Updating historical climate data can solve this limitation in future studies.

There is no doubt that models cannot predict the complexity of the real world. But to get closer to understanding this complexity, machine learning-based model like the one we have used here is vital tool in many areas of entomology and VBDs. MaxEnt modeling assumes that the available data is representative of the true distribution of the phenomenon being modeled, but this may not always be the case. To resolve this, it is important to evaluate the model's predictive performance using independent validation data and incorporate new information and data sources when available to update or refine the model. Moreover, the AUC may be overly optimistic due to the model evaluation scheme used. With goal-oriented validation strategies for spatio-temporal prediction models, the neglected problem of dependencies caused by the nature of spatio-temporal data can be addresse^48^. Furthermore, we also suggest using more than one model or ensemble models. It makes it possible to better decide which one fits best and has the best function on the distribution of species or to identify areas at risk. In addition, models and tools with interpretable machine learning such as Partial Dependence Plots (PDPs) can be used in future studies.

The map of the global occurrences of *An. stephensi* shows that the data collated cover a quite restricted geographical area (Northern-East Africa, Middle East, and South Asia). As the our model was trained with data that cover a restricted geographical area, when conducting global predictive mapping, should be taken with many care for the areas that are located far away from the training data. In order to address the mentioned limitation, care must be taken that a thorough understanding of the vector's ecology, along with its historical, recent, and current spatial distributions, should be used to inform the modeling and interpretation process. The studies have proposed different approaches and scenarios for mapping. These scenarios will have different complexities that may affect the interpretation of the resulting projections, but taking the time to consider what the observed data shows and the implications of the possible scenarios is a starting point for more accurate interpretation of the predicts maps^49^.

Finally, our practical suggestion related to current study is increasing local studies. Considering that in this study we only used the larval sampling method, this can be stated as a limitation of this study. It is better to use common mosquito sampling methods such as total catch, hand catch and stuff like that in future studies. Therefore, we suggest conducting a comprehensive study on the presence and absence of the invasive *An. stephensi* in our projected hotspot areas, which calls for more sampling for a more detailed analysis. Based on the findings and implications of the study, we would advise the NMEP to consider establishing and executing formal protocols for *An*. *stephensi* surveillance in Ghana to effectively manage the menace of malaria transmission and its dynamics. Given that the species is anthropophilic, the movement of people is more likely to be a contributing factor. Due to the morphological similarity of *An. stephenie* to *An. arabiensis*, so it would be great if the identification was based on genetic analysis.

## Conclusion

The presence and potential spread of *An. stephensi* poses a significant challenge to malaria control and elimination efforts in Africa, particularly in urban areas. The introduction of *An. stephensi* to the continent has been documented. Planning for public health and risk mitigation certainly includes evaluating the potential risk of *An. stephensi* expansion.The predicted hotspots were across several ecological zones of the country and included urban and peri-urban regions mostly influenced by human populations and mean annual temperature as the most important variable in the model. In general, the findings from the study provide important information for the surveillance and future development of control strategies as well as mitigating the spread of *An. stephensi* in Ghana. The National Malaria Elimination Program (NMEP) must consider both healthcare access and the varied geographic distribution of malaria burden in the country to increase the effectiveness of control measures.

## Methods

### Data collection and preparation

#### Occurrence data

The terms "*An. stephensi*" and "malaria" were used in a literature search of several online scientific sources (Google Scholar, PubMed, and Web of Science) from 2011 to 2022 to compile a database of the presence-only locations of the vector worldwide. From the invasive species threat map, we downloaded global occurrence data from the WHO website. A database was created in Excel with the species' presence points of geographical locations. A total of 1059 geographical points were collated, most of them occurring in Asia the species’ native continent, and Africa since its invasion (Supplementary material: Fig S1). Given the fact that the occurrence records were obtained from several data sources, we started by eliminating duplicate points. Distribution points that were close to one another (distance ≤ 5 km) were then eliminated using the spatially rarefy occurrence data tool in SDMs toolbox v2.5 via ArcGIS v10.5 to prevent pseudo-replication and spatial autocorrelation. Finally, out of the 1059 points, 660 were used in the MaxEnt model (Supplementary material: Excel file S1).

#### Environmental data

Additionally, topographic (altitude) and bioclimatic data were downloaded with a spatial resolution of approximately 5 km^2^ from the WorldClim (v2.1) database (www.worldclim.org). The environmental suitability of the *An. stephensi* vector was predicted using historical climate data (1970–2000). The model also included slope, NDVI (Normalized Difference Vegetation Index) layers, and population density grid, which were obtained from the Socioeconomic Data Applications Centre website and Google Earth Engine, respectively, and had the same resolution as the bioclimatic layers. It is widely known that many climate variables are highly correlated with each other and inclusion of highly correlated variables in statistical models can lead to spurious results. We tested all variables for correlation in ArcGIS v10.5 using the "SDMtools" tool. This tool evaluates the correlations among all input environment data through Pearson’s correlation and then removes layers that are correlated at the user-specified level. Finally, a Pearson’s correlation matrix was generated for each pair of variables. The Pearson correlation coefficient measures the linear relationship between two continuous variables. It provides a numerical value between -1 and 1, where -1 indicates a perfect negative correlation, 1 indicates a perfect positive correlation, and 0 indicates no correlation (Fig. 1).

#### Modelling

To forecast the probable environmental suitability for *An. stephensi* around the world, we used the Maximum Entropy (MaxEnt) model, which has been recognized be the most widely used among many other modelling techniques. The most recent bioclimatic data were used for the modelling in the MaxEnt program version 3.4.3^35^. The software used a jack-knife method to evaluate the importance of each variable in the model by analysing the contribution of each environmental and bioclimatic variable. The model ran ten repetitions, with training data of 80% and a random test sample of 20%. The model's output gave the best predictions for the possible distributions of the species. We then clipped using Ghana's boundary shape file after importing the model output into ArcGIS 10.5.

#### Assessing the model

The Area Under Curve (AUC) statistics, a measure that is threshold independent, was used to assess the model's performance. It is one of the most used statistical methods for evaluating models. The AUC uses values ranging from 0 to 1 to assess the model's capacity for prediction. Following the AUC measurement principle, a value of 0.5 implies a model with the least accuracy (signifying random prediction), whereas an AUC of 0.75 or higher is regarded as suitable and an AUC of 0.9 or above as excellent. Essentially, the model performs better with a higher AUC value of^30^.

#### Field sampling

We binaries the MaxEnt modeled map. The first category was 1 value, where it represents areas with a probability of presence greater than 60%. On the other hand, the second category was 0 value, which it represents areas with a presence below 60%. Indeed, communities for field sampling represented the presence areas of *An. stephensi* species with a probability of over 60%. It should be noted that to avoid sampling bias in the evaluation of the models, we randomly selected areas where no studies have been conducted regarding the presence of this species until then (Supplementary material: Fig S2). The output of the model was tested in the field, through mosquito larval sampling. During April–May 2023, ten communities (eight in the Greater Accra region and two in the Central region) were selected, for sampling. Five breeding sites were sampled in each community making a total of 50 breeding sites. Larvae were collected from typical *Anopheles* and some *Aedes* breeding areas as described in the literature^50,51^, as well as some artificial breeding places that are typical to the target species (*An. stephensi*) such as clear water in containers, household water storages, and a few air conditioning units^52^ (Supplementary material: Fig. S3). A global positioning system (GPS) device was used to record the coordinates for each breeding site. We also recorded geographic coordinates of sites from which sampling was done, as reference points for mapping species presence. The larval samples were transported in improvised breeder cups to the Noguchi Memorial Institute for Medical Research (NMIMR) and reared to adult mosquitoes in the Institute’s insectary. The adult mosquitoes, reared from the field-collected larvae, were aspirated into holding cups and knocked down in a −20 °C freezer for identification. They were then sorted into Anophelines and Culicines*.* Each *Anopheles* mosquito was put into a tube for confirmation. All *Anopheles* mosquitoes were morphologically identified using an identification key^45^.

### Ethical approval

This study was conducted under the ethical principles, national norms, and standards for conducting Medical Research in Iran. The Research Ethics Committees of the School of Public Health & Allied Medical Sciences-Tehran University of Medical Sciences approved this project under code: IR.TUMS.SPH.REC.1402.032.

### Supplementary Information

Supplementary Information 1.

Supplementary Information 2.

## Data Availability

All data needed to evaluate the conclusions in the paper are present in the paper, or the references cited here within.
