# Supplementary material for: Predicting the environmental suitability for Anopheles stephensi under the current conditions in Ghana
Source: Sci Rep. 2024 Jan 11;14:1116. doi: 10.1038/s41598-024-51780-7 (PMC10784561; doi:10.1038/s41598-024-51780-7)
Supplement: Supplementary file 1 — Supplementary Information 1. [file 41598_2024_51780_MOESM1_ESM.docx]

**Supplementary Information**

**Predicting the environmental suitability for *Anopheles stephensi* under the current conditions in Ghana**

Rahmat Bint Yusif Ismail, Faramarz Bozorg-Omid, Joseph Harold Nyarko Osei, Sellase Pi-Bansa, Kwadwo Kyeremeh Frempong, Mavis Koryo Ofei, Helena Anokyewaa Boakye, Jane Ansah-Owusu, Sandra-Candys Adwirba Akorful, Christopher Nii Laryea Tawiah-Mensah, Mufeez Abudu, Andy Asafu-Adjaye, Maxwell Alexander Appawu, Daniel Adjei Boakye, Hassan Vatandoost, Mohammad Mehdi Sedaghat, Fahimeh Youssefi, Ahmad Ali Hanafi-Bojd and Samuel Kweku Dadzie

**Figures S1-S3**


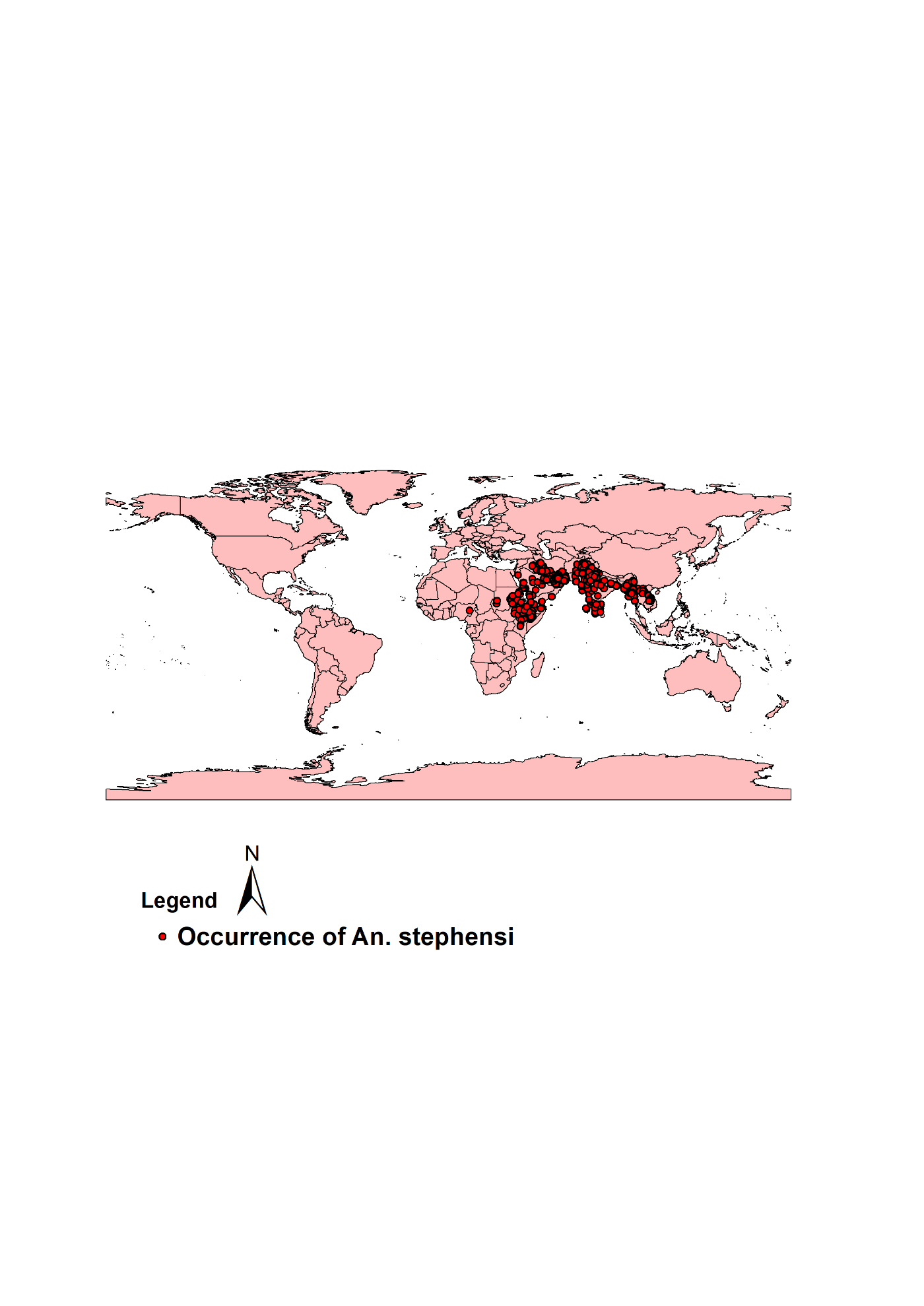


**Supplementary Fig S1.** Global occurrence points of *Anopheles stephensi*, 2011-2022. The Map was generated using ArcGIS v10.5 (www.esri.com).


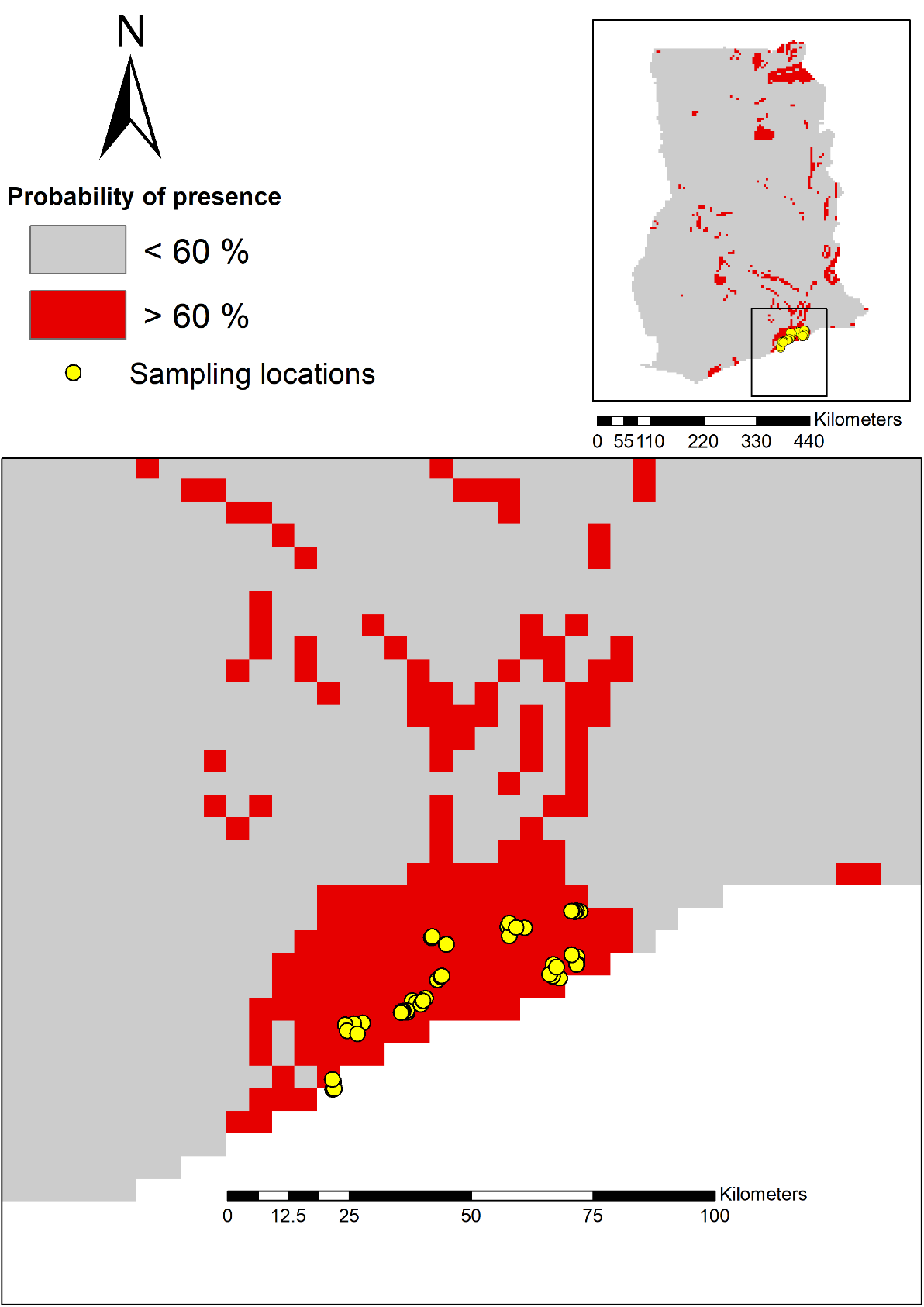


**Supplementary Fig S2.** The location of the selected districts to collect mosquito species with presence probability < 60 % in Greater Accra Province, Ghana. The Map was generated using ArcGIS v10.5 (www.esri.com).


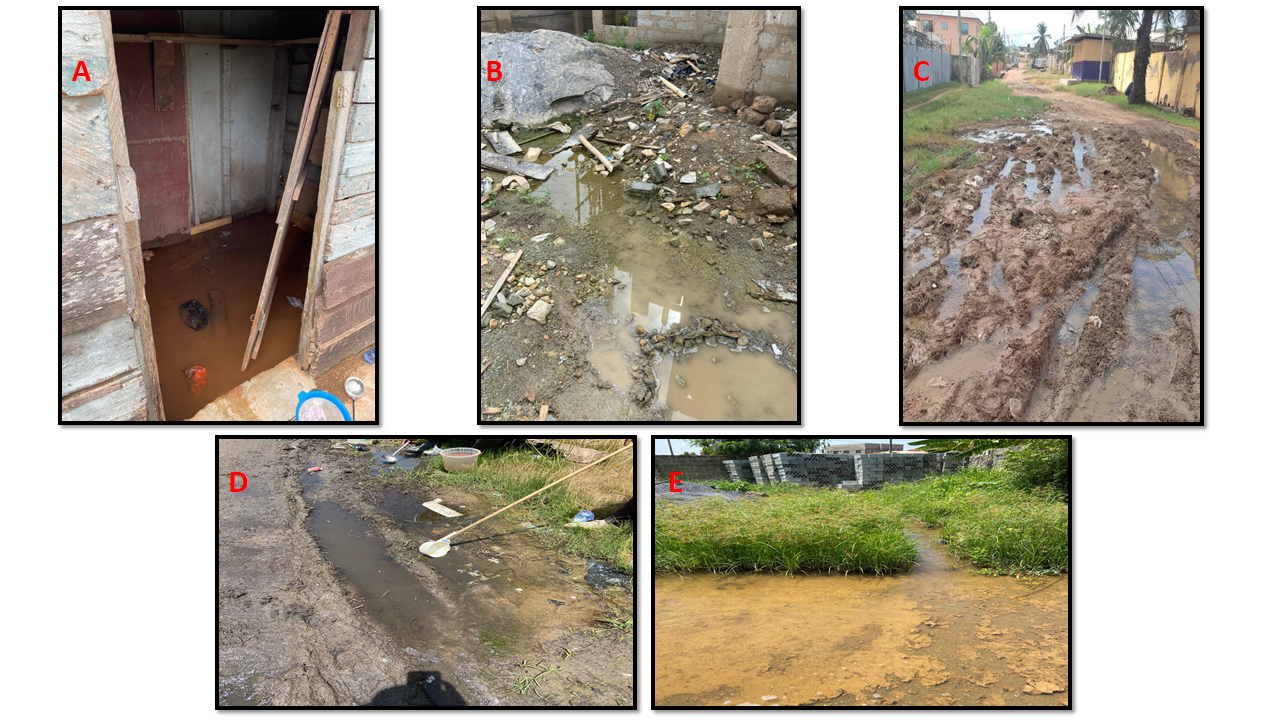


**Supplementary Fig S3.** Different classes of larval habitats in the sampling sites in Greater Accra Province, Ghana. A) Man-made (artificial) containers B, C, D) Semi-natural water bodies (puddles and road tracks) E) Semi-permanent surface water.
